# Amarogentin, a Secoiridoid Glycoside, Abrogates Platelet Activation through PLC****γ**2**-PKC and MAPK Pathways 

**DOI:** 10.1155/2014/728019

**Published:** 2014-04-29

**Authors:** Ting-Lin Yen, Wan-Jung Lu, Li-Ming Lien, Philip Aloysius Thomas, Tzu-Yin Lee, Hou-Chang Chiu, Joen-Rong Sheu, Kuan-Hung Lin

**Affiliations:** ^1^Graduate Institute of Medical Sciences and Department of Pharmacology, College of Medicine, Taipei Medical University, 250 Wu-Hsing Street, Taipei 110, Taiwan; ^2^School of Medicine, Taipei Medical University, 250 Wu-Hsing Street, Taipei 110, Taiwan; ^3^Department of Neurology, Shin Kong Wu Ho-Su Memorial Hospital, 95 Wen-Chang Road, Taipei 111, Taiwan; ^4^Department of Microbiology, Institute of Ophthalmology, Joseph Eye Hospital, 138 Melapudur, Tiruchirappalli, Tamil Nadu 620 001, India; ^5^College of Medicine, Fu-Jen Catholic University, 510 Zhongzheng Road, New Taipei City 242, Taiwan; ^6^Central Laboratory, Shin-Kong Wu Ho-Su Memorial Hospital, 95 Wen-Chang Road, Taipei 111, Taiwan

## Abstract

Amarogentin, an active principle of *Gentiana lutea*, possess antitumorigenic, antidiabetic, and antioxidative properties. Activation of platelets is associated with intravascular thrombosis and cardiovascular diseases. The present study examined the effects of amarogentin on platelet activation. Amarogentin treatment (15~60 **μ**M) inhibited platelet aggregation induced by collagen, but not thrombin, arachidonic acid, and U46619. Amarogentin inhibited collagen-induced phosphorylation of phospholipase C (PLC)**γ**2, protein kinase C (PKC), and mitogen-activated protein kinases (MAPKs). It also inhibits *in vivo* thrombus formation in mice. In addition, neither the guanylate cyclase inhibitor ODQ nor the adenylate cyclase inhibitor SQ22536 affected the amarogentin-mediated inhibition of platelet aggregation, which suggests that amarogentin does not regulate the levels of cyclic AMP and cyclic GMP. In conclusion, amarogentin prevents platelet activation through the inhibition of PLC**γ**2-PKC cascade and MAPK pathway. Our findings suggest that amarogentin may offer therapeutic potential for preventing or treating thromboembolic disorders.

## 1. Introduction

*Gentiana lutea* is a plant that belongs to the family Gentianaceae, which grows in the mountains of central and southern Europe and in western Asia [1]. It is commonly used to treat digestive diseases. The extract of this plant was reported to inhibit cell proliferation of vascular smooth muscle cells [2] and exhibit antioxidant and radioprotective activities [3, 4]. It contains some of the most bitter-tasting compounds known and is used as a scientific basis for the measurement of bitterness. The active principle amarogentin, which is isolated from the extract of* Gentiana lutea*, is a bitter-tasting secoiridoid glycoside that was found to activate the human bitter taste receptor hTAS2R50 [5]. Amarogentin has also been reported to possess antitumorigenic [6, 7] and antidiabetic activities [8].

It is well-known that blood platelets play important roles in haemostatic processes and wound repair. When blood vessels are damaged, blood platelets will form platelet plugs on the sites of vessel injury in order to prevent blood loss [9]. However, deregulation of platelet activity may cause a wide variety of cardiovascular diseases, such as intraluminal thrombosis and atherosclerosis. Therefore, the development of antiplatelet agents that can prevent heart attack and ischemic stroke is warranted.

Although previous studies have suggested that amarogentin effectively prevents vascular diseases, its effects on platelet activation and thrombosis remains unclear. Since our preliminary study showed that amarogentin (15~60 *μ*M) inhibits collagen-induced platelet aggregation in human platelets, we further systematically investigated the detailed mechanisms underlying the amarogentin-mediated inhibition of platelet activation.

## 2. Materials and Methods

### 2.1. Materials

Amarogentin (88.9%) was purchased from ChromaDex (Irvine, CA). Arachidonic acid (AA), collagen (type I), 9,11-dideoxy-11*α*,9*α*-epoxymethanoprostaglandin (U46619), luciferin-luciferase, thrombin, SQ22536, phorbol-12,13-dibutyrate (PDBu), and 1H-[1,2,4]qxadiazolo[4,3-a]quinoxalin-1-one (ODQ) were purchased from Sigma (St. Louis, MO). The anti-phospho-c-Jun N-terminal kinase (JNK) (Thr^183^/Tyr^185^) and anti-phospho-p38 mitogen-activated protein kinase (MAPK) monoclonal antibodies (mAbs), and the anti-phospho-p44/p42 extracellular signal-regulated kinase (ERK) (Thr^202^/Tyr^204^), anti-phospholipase C*γ*2 (PLC*γ*2), and anti-phospho (Tyr^759^) PLC*γ*2 polyclonal antibodies (pAbs) were purchased from Cell Signaling (Beverly, MA). The anti-phospho-p38 MAPK Ser^182^ mAb was purchased from Santa Cruz (Santa Cruz, CA). The anti-*α*-tubulin mAb was purchased from NeoMarkers (Fremont, CA). The anti-phospho-Akt (Ser^473^) and anti-Akt mAbs were purchased from Biovision (Mountain View, CA). The horseradish peroxidase- (HRP-) conjugated donkey anti-rabbit immunoglobulin G (IgG), the Hybond-P polyvinylidene difluoride (PVDF) membrane, the sheep anti-mouse IgG, and the enhanced chemiluminescence western blotting detection reagent were purchased from Amersham (Buckinghamshire, UK). The amarogentin was dissolved in DMSO and stored at 4°C.

### 2.2. Platelet Aggregation and ATP Release

The methods described by Sheu et al. [10] and Lin et al. [11] were followed for the preparation of human platelet suspensions. Blood was collected from healthy human volunteers (informed consent) who did not take medication during the preceding 2 wk and was mixed with acid-citrate-dextrose solution (1 : 9). The blood samples were subjected to centrifugation at 120 g for 10 min, and platelet-rich plasma (PRP) was collected. PRP was supplemented with PGE_1_ (0.5 *μ*M) and heparin (6.4 IU/mL) and then incubated for 10 min at 37°C. After centrifugation at 500 g for 10 min, the platelet pellets were suspended in Tyrode's solution containing 3.5 mg/mL bovine serum albumin (BSA), pH 7.3 (NaCl 11.9 mM, KCl 2.7 mM, MgCl_2_ 2.1 mM, NaH_2_PO_4_ 0.4 mM, NaHCO_3_ 11.9 mM, and glucose 11.1 mM). Then, PGE_1_ (0.5 *μ*M), apyrase (1.0 U/mL), and heparin (6.4 IU/mL) were added, and the mixture was incubated for 10 min at 37°C. The mixtures were centrifuged at 500 g for 10 min and subjected for the repeated washing procedure. Finally, the platelet pellets were resuspended by Tyrode's solution, and then calcium chloride was added to platelet suspensions in which the concentration of Ca^2+^ was 1 mM. This study wasapproved by the Institutional Review Board of Taipei Medical University and conformed to the directives of the Helsinki Declaration.

As previously described [10, 11], platelet aggregation was measured according to the turbidity of platelet suspensions and recorded by a Lumi-Aggregometer (Payton Associates, Scarborough, ON, Canada). Before the addition of agonists to induce platelet aggregation, the platelet suspensions (3.6 × 10^8^cells/mL) were pretreated with various concentrations of amarogentin or an isovolumetric solvent control (0.5% DMSO) for 3 min. Light-transmission unit was used to present the extent of platelet aggregation. For the measurement of ATP release, a 20 *μ*L of luciferin-luciferase mixture was added 1 min before adding amarogentin or agonists, and the relative amount of ATP release was compared to the solvent control.

### 2.3. Western Blotting

Western blotting assay was performed as described previously [11]. Briefly, after pretreatment of various concentrations of amarogentin, agonists were added to the washed platelets (1.2 × 10^9^ cells/mL) for the indicated time to trigger platelet activation. The platelet pellets were collected after centrifugation and then immediately lysed in extraction buffer. Samples containing 80 *μ*g of protein were subjected to sodium dodecyl sulfate polyacrylamide gel electrophoresis (SDS-PAGE, 10–12%) and electrotransferred onto the polyvinylidene difluoride (PVDF) membranes by Bio-Rad semidry transfer unit (Hercules, CA). Blots were blocked with 5% BSA in TBST (10 mM Tris-base, 100 mM NaCl, and 0.01% Tween 20) for 1 h and probed with the various primary antibodies (1 : 1000) for 2 h. The membranes were incubated with horseradish peroxidase- (HRP-) conjugated anti-mouse IgG or anti-rabbit IgG (1 : 3000) for 1 h. Immunoreactivity was detected by an enhanced chemiluminescence system. The quantitative data were obtained by scanning the reactive bands and quantifying the optical density using a computing densitometer and Bio-profil Biolight software, version V2000.01 (Vilber Lourmat, Marne-la-Vallée, France).

### 2.4. Fluorescein-Induced Thrombus Formation in the Microvessels of Mouse Mesentery

The protocols conformed to the Guide for the Care and Use of Laboratory Animals (NIH publication no. 85-23, 1996). The methods to measure thrombus formation was performed as described previously [11, 12]. In brief, an external jugular vein was cannulated with a PE-10 to intravenously administer the dye and drugs after mice were anesthetized. Venules (30–40 *μ*m) were selected under a microscope. After administering 15 *μ*g/kg sodium fluorescein, the selected venules were irradiated at wavelengths below 520 nm to produce a microthrombus, and the time required to occlude the microvessel as a result of thrombus formation (occlusion time) was recorded. In this study, 9 and 18 mg/kg amarogentin were administered to evaluate its antithrombotic effects.

### 2.5. Statistical Analysis

The results are presented as the mean ± SEM at least 3 independent experiments (*n* = 3). Each experiment performs with blood from different donors. For the* in vivo* study, Paired Student's *t*-test was used to evaluate the differences of the occlusion time in the same mouse. For the* in vitro* study, if appropriate, the one-way analysis of variance (ANOVA) followed by the Student-Newman-Keuls test was used to determine the statistical differences among groups. *P* < 0.05 was considered statistically significant. Statistical analyses were performed using SAS, version 9.2 (SAS Inc., Cary, NC).

## 3. Results

### 3.1. Amarogentin Inhibits Platelet Aggregation and ATP Release

**Figure 1:**
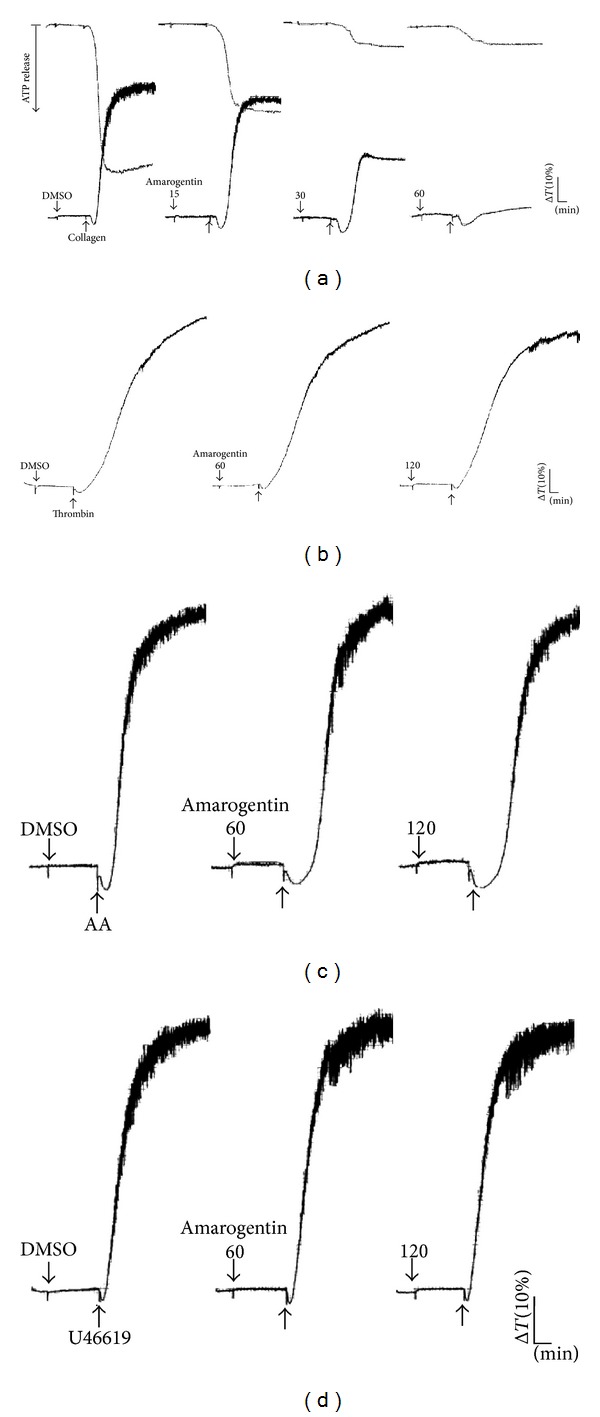
Amarogentin inhibits collagen-induced platelet aggregation and ATP release. Washed platelets (3.6 × 10^8^ cells/mL) were incubated with solvent control (0.5% DMSO) or amarogentin (15 *μ*M~120 *μ*M) for 3 min in an aggregometer cuvette. Then, 1 *μ*g/mL collagen (a), 0.01 IU/mL thrombin (b), 60 *μ*M arachidonic acid (AA) (c), or 1 *μ*M U46619 (d) was added to induce platelet aggregation and ATP-release ((a), upper tracings) for 6 min. Profiles ((a)–(d)) are representative of 3 independent experiments.

Amarogentin (15~60 *μ*M) showed more potent activity in inhibiting platelet aggregation and ATP release induced by collagen (1 *μ*g/mL) (Figure 1(a)). However, amarogentin (120 *μ*M) did not affect platelet aggregation triggered by 0.01 IU/mL thrombin, 60 *μ*M AA, or 1 *μ*M U46619 (Figures 1(b)–1(d)). The half maximal inhibitory concentration (IC_50_) of amarogentin for platelet aggregation induced by collagen was approximately 30 *μ*M. Moreover, the solvent control (0.5% DMSO) did not affect platelet activity (data not shown). The agonist collagen was used to investigate the antiplatelet mechanisms of amarogentin.

### 3.2. Effects of Amarogentin on the Phosphorylation of PLC*γ*2 and p47

**Figure 2:**
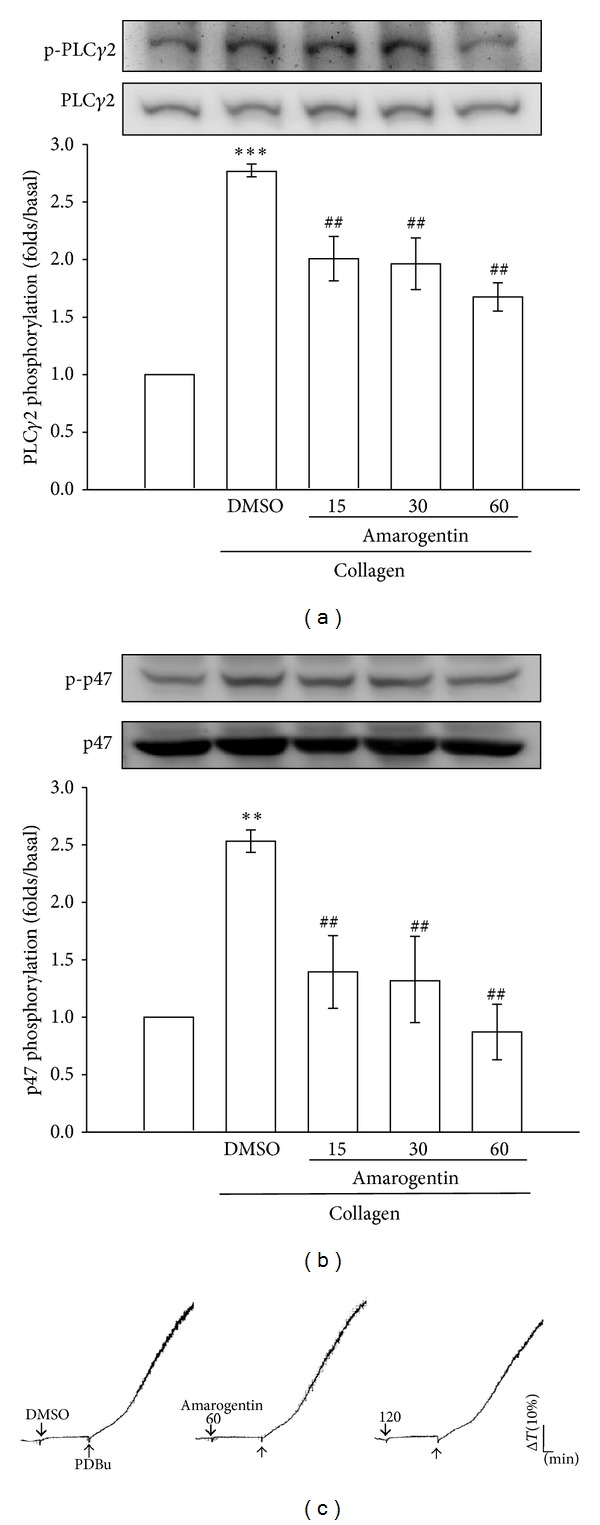
Effects of amarogentin on collagen-induced PLC*γ*2 and PKC activation. Washed platelets (1.2 × 10^9^ cells/mL) were incubated with solvent control (0.5% DMSO) or amarogentin (15 *μ*M~120 *μ*M) for 3 min. Then, 1 *μ*g/mL collagen was added to induce the phosphorylation of PLC*γ*2 (a) and p47 (b) for 5 min and 10 min, respectively. ((a) and (b)) The subcellular extracts were analyzed for PLC*γ*2 (a) and p47 (b) by western blotting. (c) Washed platelets (3.6 × 10^8^ cells/mL) were incubated with solvent control (0.5% DMSO) or amarogentin (15 *μ*M~120 *μ*M) for 3 min and then treated with 150 nM PDBu to induce platelet aggregation. Data ((a) and (b)) are presented as the mean ± SEM (*n* = 3). **P < 0.01 and ***P < 0.001, compared with the solvent control group (resting); ^##^ *P* < 0.01, compared with the positive control group (collagen only). Profiles (c) are representative of 3 independent experiments.

The PLC enzyme hydrolyzes phosphatidylinositol 4,5-bisphosphate (PIP_2_) to generate diacylglycerol (DAG) and inositol 1,4,5-trisphosphate (IP_3_). Subsequently, DAG triggers PKC activation, thereby leading to protein phosphorylation and ATP release in agonist-stimulated platelets [13]. Then, PKC phosphorylates p47 protein (pleckstrin) [13]. While platelets were treated with amarogentin, it attenuated the phosphorylation of PLC*γ*2 and p47 triggered by collagen in a concentration (15~60 *μ*M)-dependent manner (Figures 2(a) and 2(b)) but did not affect PDBu-induced platelet aggregation (Figure 2(c)).

### 3.3. Effects of Amarogentin on the Phosphorylation of MAPKs and Akt

**Figure 3:**
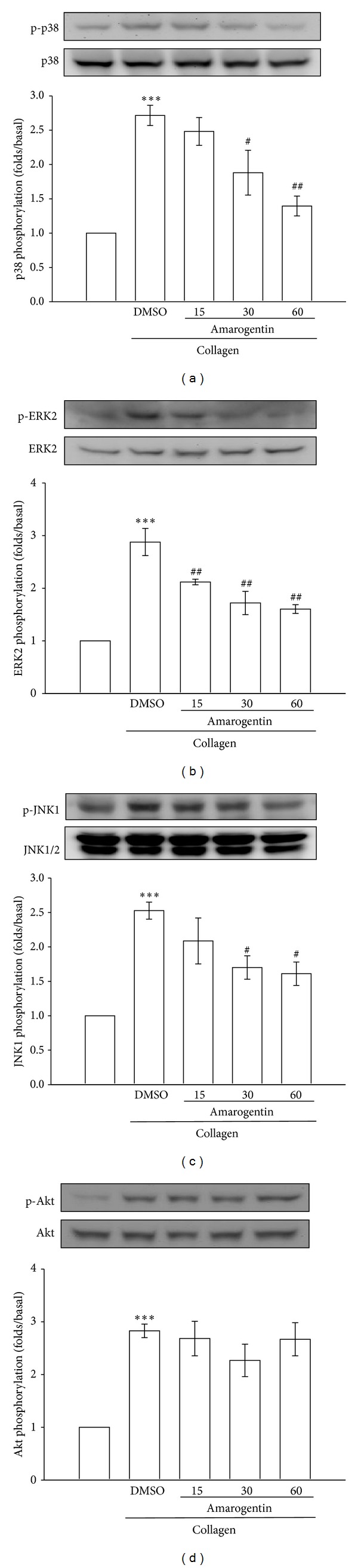
Effects of amarogentin on the phosphorylation of MAPK and Akt induced by collagen in human platelets. Washed platelets (1.2 × 10^9^ cells/mL) were incubated with solvent control (0.5% DMSO) or amarogentin (15 *μ*M~60 *μ*M) and then treated with 1 *μ*g/mL collagen to induce platelet activation. The subcellular extracts were analyzed for the phosphorylation of p38 (a), ERK2 (b), JNK1 (c), and Akt (d) by western blotting. Data are presented as the mean ± SEM (*n* = 3). ***P < 0.001, compared with the solvent control group (resting); ^#^ *P* < 0.05 and ^##^ *P* < 0.01, compared with the positive control group (collagen only).

Previous studieshave suggested that MAPKs and Akt are involved in platelet activation and thrombosis [14, 15]. Thus, we determined these signaling molecules in collagen-activated platelets to investigate the antiplatelet mechanisms of amarogentin. We found that amarogentin concentration dependently (30~60 *μ*M) inhibited collagen-induced phosphorylation of p38, ERK2, and JNK1 (Figures 3(a)–3(c)) but did not affect the phosphorylation of Akt (Figure 3(d)). These findings suggest that amarogentin inhibits collagen-induced platelet activation via MAPKs, but not Akt.

### 3.4. Effects of Amarogentin on Cyclic Nucleotides in Human Platelets

**Figure 4:**
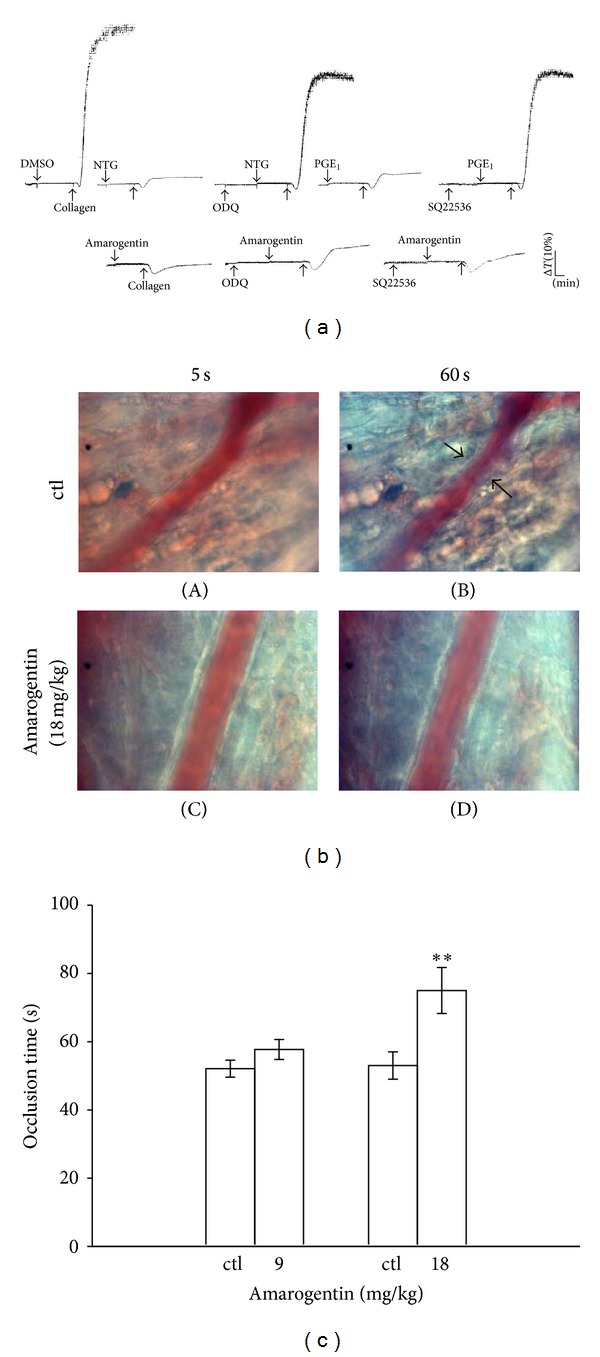
Effects of amarogentin on cyclic nucleotides in human platelets and on thrombus formation in mice. (a) Washed platelets (3.6 × 10^8^ cells/mL) were preincubated with 10 *μ*M nitroglycerin (NTG), 0.5 nM prostaglandin E_1_ (PGE_1_), or 60 *μ*M amarogentin with or without 10 *μ*M ODQ or 100 *μ*M SQ22536, followed by treatment with 1 *μ*g/mL collagen to induce platelet aggregation. ((b) and (c)) Mice were administered 0.5% DMSO (solvent control; ctl) and amarogentin (9- or 18 mg/kg). Then, mesenteric venules were selected and irradiated to induce microthrombus formation. The data in the bar graphs are presented as the mean ± SEM of the occlusion time which is seconds (*n* = 5). **P < 0.01 compared with the individual solvent control group (ctl).

As shown in Figure 4(a), both ODQ (10 *μ*M) and SQ22536 (100 *μ*M) significantly reversed the inhibition of platelet aggregation mediated by nitroglycerin (10 *μ*M)- and PGE_1_ (0.5 nM), respectively. However, these inhibitors do not reverse the amarogentin (60 *μ*M)-mediated inhibition of collagen-induced platelet aggregation, which indicates that cyclic AMP (cAMP) and cyclic GMP (cGMP) are not involved in the antiplatelet effects of amarogentin.

### 3.5. Effects of Amarogentin on Thrombus Formation in Mice

For the* in vivo* study, fluorescein sodium (15 *μ*g/kg) was intravenously administrated and irradiated to induce thrombus formation in the mesenteric microvessels of mice and the time of occlusion was found to be approximated at 60 s (Figure 4(b)). Treatment of mice with 18 mg/kg amarogentin prolonged the occlusion time (75.2 ± 6.7 s) of thrombus formation, compared with the solvent control (53.0 ± 4.0 s) (Figure 4(c)).

## 4. Discussion

We investigated the effect of amarogentin, an active principle of* Gentiana lutea*, on platelet activation* in vitro* and thrombus formation in a mouse model. In the present study, we demonstrated for the first time that amarogentin inhibits platelet activation* in vitro* via inhibiting PLC*γ*2-PKC/MAPK cascade and* in vivo *through reversing thethrombus formation. Our results revealed that amarogentin concentration dependently inhibited collagen-induced platelet activation. Moreover, amarogentin did not affect the responses stimulated by AA, U46619, and thrombin in human platelets. These findings indicate that amarogentin mainly inhibits collagen-induced platelet activation.

GPVI, a member of the immunoglobulin superfamily, is required for collagen-induced platelet activation [9]. When platelets are exposed to collagen, a signaling complex, including LAT, SLP-76, and Gads, activates PLC*γ*2, leading to PKC activation and Ca^2+^ release [9]. In the present study, we found that amarogentin could inhibit the phosphorylation of PLC*γ*2 and PKC. However, amarogentin did not affect the PKC activator PDBu-stimulated platelet aggregation, indicating that amarogentin may act on the upstream of PKC.

It is well established that MAPKs, including ERKs, JNKs, and p38, have been identified in platelets [16], where they are activated by collagen and thrombin, and are involved in thrombosis [14]. The ERK and p38 play important roles in stimulating the secretion of granules and facilitating clot retraction [17]. During platelet activation, the AA metabolism may offer a positive feedback amplifier to activate p38, followed by the stimulation of cytosolic phospholipase A_2_, which promotes thromboxane A_2_ formation [18]. In addition, JNK1 is reportedly involved in collagen-induced platelet aggregation and thrombus formation [19]. The time of thrombus formation was significantly prolonged in JNK1^−/−^ arterioles in an* in vivo* model and platelet secretion was impaired in JNK1^−/−^ platelets* in vitro *[20]. In this study, we demonstrated that the activation of MAPKs is inhibited by amarogentin, suggesting that amarogentin attenuated platelet activation and thrombus formation, at least in part, through MAPK cell-signaling pathway. In addition, several studies showed that PI3K/Akt plays an important role in regulating platelet aggregation and thrombus formation [15, 21, 22]. Thus, we also observed the influence of amarogentin on Akt and found that Akt was not associated with amarogentin-mediated inhibition of platelet activation.

cAMP and cGMP have been known to inhibit many aspects of platelet activation, including Ca^2+^ release, G-protein activation, granule release, and platelet adhesion and aggregation [23]. cAMP and cGMP strongly attenuate the elevation of cytosolic Ca^2+^ concentrations, at least in part, via phosphorylating IP_3_ receptor, and are also reported to block p38 activation in platelets [23]. We found that SQ22536 and ODQ did not reverse the amarogentin-mediated inhibition of platelet aggregation. These results revealed that amarogentin did not regulate the level of cAMP and cGMP.

**Figure 5:**
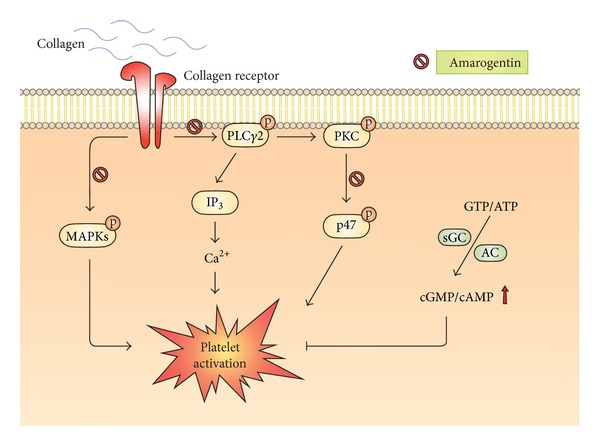
Hypothesis regarding the inhibitory signaling of amarogentin in platelet activation. Amarogentin may inhibit both the PLC*γ*2-PKC-p47 cascades and MAPK signaling pathway, ultimately inhibiting platelet activation. DAG: diacylglycerol; IP_3_: inositol 1,4,5-trisphosphate.

In conclusion, we demonstrated that amarogentin abrogates platelet activation probably via inhibiting the PLC*γ*2-PKC-p47 cascades and MAPK signaling pathway (Figure 5), finally reducing thrombus formation. Our findings suggest that amarogentin may provide therapeutic potential for preventing or treating thromboembolic disorders.
